# Cross-Sensor Fingerprint Matching Using Siamese Network and Adversarial Learning

**DOI:** 10.3390/s21113657

**Published:** 2021-05-24

**Authors:** Adhwa Alrashidi, Ashwaq Alotaibi, Muhammad Hussain, Helala AlShehri, Hatim A. AboAlSamh, George Bebis

**Affiliations:** 1 Department of Computer Science, CCIS, King Saud University, Riyadh 11451, Saudi Arabia; 437202899@student.ksu.edu.sa (A.A.); 438204233@student.ksu.edu.sa (A.A.); hatim@ksu.edu.sa (H.A.A.); 2 Computer Science and Engineering Department, Jubail University College, Al Jubail 35716, Saudi Arabia; SHEHRIH2@ucj.edu.sa; 3 Department of Computer Science and Engineering, University of Nevada, Reno, NV 89557, USA; bebis@unr.edu

**Keywords:** biometrics, Siamese network, cross-sensor fingerprint matching, CNN, adversarial learning, GAN

## Abstract

The fingerprint is one of the leading biometric modalities that is used worldwide for authenticating the identity of persons. Over time, a lot of research has been conducted to develop automatic fingerprint verification techniques. However, due to different authentication needs, the use of different sensors and the fingerprint verification systems encounter cross-sensor matching or sensor interoperability challenges, where different sensors are used for the enrollment and query phases. The challenge is to develop an efficient, robust and automatic system for cross-sensor matching. This paper proposes a new cross-matching system (SiameseFinger) using the Siamese network that takes the features extracted using the Gabor-HoG descriptor. The proposed Siamese network is trained using adversarial learning. The SiameseFinger was evaluated on two benchmark public datasets FingerPass and MOLF. The results of the experiments presented in this paper indicate that SiameseFinger achieves a comparable performance with that of the state-of-the-art methods.

## 1. Introduction

The use of fingerprint modality as a means of person verification has been on the rise over the past many years. Different law enforcement institutions, security agencies, and various service providers have developed huge databases, usually with a specific sensor for verification purposes. Moreover, as a result of recent technological advancements, the development of numerous low-cost and smart fingerprint sensors, embedded on devices such as smart-phones and PCs, has been identified globally. Because of this, the fingerprints can now be acquired using different sensors for the enrollment and verification phases. Most of the existing techniques were designed with the hypothesis that the same sensor is used for enrollment of fingerprints in a database and for the query to authenticate the identity of a person. This kind of fingerprint matching is known as regular matching. The performance of such a method is high when the same sensor is used for the enrollment and verification phases, but its performance degrades significantly when a different sensor is used for each phase. This gives rise to the cross-sensor or sensor interoperability problem that is, different sensors are used for enrollment and query stages of a fingerprint recognition system. The fingerprint sensors depend on several technologies such as ultra-sound and solid-state, and each sensor has its own type of technology that differs from others; this difference makes the interoperability problem even more exigent [1]. Recent studies [2] indicate that there is a need for improvements to achieve better cross-device fingerprint verification performance and have highlighted the need to conduct research on fingerprint sensor interoperability problem [2,3]. However, this problem has been studied by a few researchers. The existing methods are based on hand-engineered features [4], nonlinear distortions [5,6], and scaling of fingerprints [7,8,9]. Despite these efforts, the interoperability problem is still challenging. In recent years, deep learning methods, which use deep artificial neural network architectures, have shown a remarkable success for image classification, recognition, and feature representation. However, only few research methods have been proposed to overcome fingerprint sensor interoperability problem using deep learning methods [10,11]. This observation motivated us to propose a new method based on deep learning and explore its effect on the cross-matching problem. In view of this, we introduce a cross-sensor matching method using Siamese network trained with adversarial learning [4]. The Siamese network, adopting the convolutional neural network as a backbone, is trained with Gabor-HoG descriptor [4] to learn the correspondences. The Gabor-HoG descriptor which encodes multi-scale local ridge structures and changes across ridge patterns. The proposed method employs a simple architecture for Siamese network and train it using adversarial learning. The feature vectors generated from the matching pair of fingerprints with the Siamese network are used to predicts the probability of whether the fingerprint pair matches or does not match. The training of a Siamese network is a challenging task, we used adversarial learning for its training. For evaluation of the proposed method, we performed experiments on two public benchmark databases (FingerPass [12], MOLF [13]), and compared results with the state-of-the-art methods. Specifically, our contributions in this paper can be summarized as follows:

-  A novel deep learning method has been introduced to address the cross-matching problem. The method is alignment free, which reduces the matching time of fingerprints.
-  A Siamese network has been proposed to learn fingerprint feature correspondences. The architecture of the Siamese network has been designed specifically to address the cross-matching problem.
-  An adversarial learning method has been used to train the Siamese network.
-  The method has been evaluated comprehensively using two benchmark datasets and compared with state-of-the-art methods.

The rest of the paper is organized as follows: Section 1 presents a brief review of the state-of-the-art methods on fingerprint sensor interoperability problem, and Section 2 gives the detail of the proposed method, which we call *fingerprint matching with Siamese network* (*SiameseFinger*). Section 4 gives details of the experiments, results and discussion. The conclusion and future work are presented in Section 5.

### Related Work

The problem of cross-sensor matching has been addressed by a number of researchers because of its importance. The proposed solutions can be categorized into two main groups: methods based on hand-engineered features and deep learning methods.

Some methods based on hand-engineered features employ traditional features such as: Gabor-HoG, Co-occurrence of ridge orientations (Co-RoR), and local binary patterns (LBPs) [14]. Helala et al. [4] introduced the crossVFinger method to solve the cross-sensor fingerprint matching; they designed a novel feature descriptor called Co-occurrence of ridge orientations (Co-RoR)to take into consideration the ridge orientation field for increasing the robustness of the crossVFinger. The Co-RoR fused with Gabor-HoG descriptor using Canonical Correlation Analysis(CCA). This method improves the execution time by matching fingerprints without the need of the registration process. Further, Helala et al. [2] fused three types of descriptors: Gabor HoG, Binary Gradient Pattern (BGP), and orientations using a simple weighted sum to represent a fingerprint. The comparison of results shows that this method gets good result. Though this method overcomes of cross-matching problem partially, it is still not robust against sensors of different technology types. Ross and Nadgir [15] employed the thin-plate spline model (TPS) and a non-linear calibration scheme to register a pair of fingerprints captured with different sensors. This method is effective to deal with the variations of inter-sensor geometric features. This model uses a calibration technique considering features extracted from pairs of few fingerprints and its generalization to all possible variations in fingerprints across different sensors is not clear. Lugini et al. [16] considered a statistical standpoint to propose a solution for the sensor interoperability problem. They used different sensors for enrollment and verification, and calculated the levels of match scores based on their change across different sensors. For evaluation, they used as a large database captured from 494 subjects; it contains scanned ink-based fingerprints as well as the fingerprints captured with four different sensors. They concluded that the diversity of the sensors affects the false non-match rates, but it has no effect on false match rates of fingerprint matching systems. Mason et al. [17] represented a fingerprint by fusing a number of selected features such as: device ID, quality measures, average gray level, photo response non-uniformity (PRNU) noise, minutia count, mean of the orientation coherence matrix, contrast and first-order statistics. In addition, they used the attributes such as match scores and alignment parameters extracted from pairs of fingerprints. For matching, they employed a tree-based method.

Very few researchers used deep learning for solving the sensor interoperability problem. Lin and Kumar [10] considered the problem of fingerprint interoperability between contactless and contact-based sensors, and designed a multi-Siamese based on convolutional neural network (CNN), which takes a pair of fingerprints as input, computes minutiae and ridge map from each fingerprint, processes them through different convolutional and fully connected layers to extract deep fingerprint representation. The network was trained using a distance-aware loss function. This method was evaluated using two public domain databases, which consist of contact-based fingerprints and the corresponding contactless fingerprints. Lin and Kumar [18] also investigated the potential of using CNN to precisely match contact-based and contactless fingerprint images through employing two phases: preprocessing and recognition. The input fingerprint is first enhanced in the preprocessing phase. In the recognition phase, the enhanced image is matched with the fingerprint stored in the database.

Although, some of the above methods give acceptable results, their generalization to different types of sensors is not clear. None of the above methods employed deep learning and adversarial learning based Siamese network for contact-based cross-sensor matching. It hypothesized that a Siamsese network based a CNN model and adversarial can produce better results for cross-sensor fingering matching, because it can learn to reduce the intra-class variation of fingerings captured with different sensors using adversarial learning.

## 2. Proposed Method

**Figure 1:**
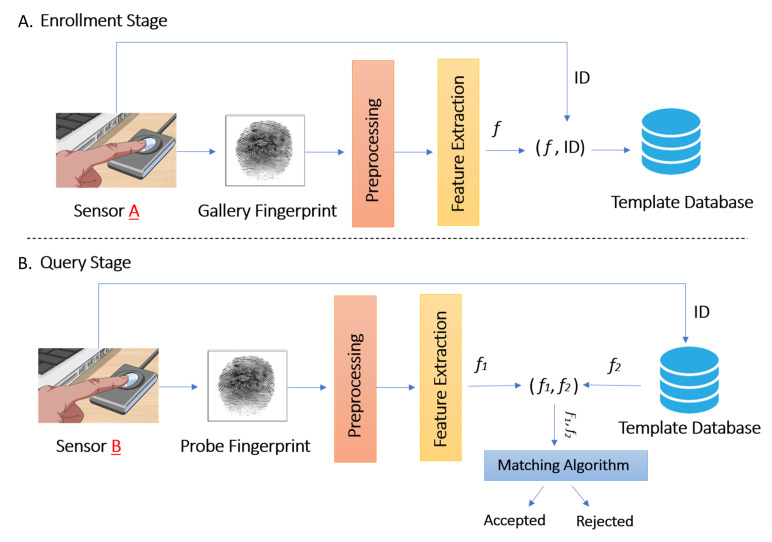
Cross-sensor fingerprint matching system.

An overview of the proposed system for cross-sensor fingerprint matching is shown in Figure 1. During the enrollment stage, sensor A is used to capture fingerprints, which are preprocessed using the method proposed by Hong et al. [15]. From the preprocessed fingerprints, the features are extracted using the Gabor-HoG descriptor [4] and stored with the corresponding IDs in the template database. In the query stage, a fingerprint is captured using sensor B, it is pre-processed using the same method and the features are extracted using the same descriptor used in the enrollment stage. Then the similarity between features S1 of the query fingerprint captured from sensor B and the features S2 retrieved from the template database by the user ID is calculated to decide whether there is a “match” or “non-match”; it is a matching problem, which is formulated in the following paragraph.

### 2.1. Problem Formulation

The problem is to match the fingerprints of a subject captured with two different sensors. Assume that there are N subjects, and K fingerprints are captured from each subject with each of two different sensors; the fingerprints of the same finger differ in resolution and fine-detail depending on the technology type of each sensor. Let $F_{s}=\left\{F_{ij}^{s}|1\leq i\leq K,\;1\leq j\leq N\right\},\;\;s=1,\;2$ be the two sets of feature vectors extracted using Gabor-HoG descriptor from fingerprints of N subjects captured with two sensors, where $F_{ij}^{s}$ represents the feature vector of *i*th fingerprint of *j*th subject captured with *s*th sensor such that $F_{ij}^{1}$∈$R^{d}$ and $F_{ij}^{2}$∈$R^{d}$, that is, the feature vectors extracted from fingerprints captured from each sensor have same dimension. Assume that $F_{1}$ represents the gallery set (captured with sensor A) and $F_{2}$ is the probe set (captured with set B). The set $F_{1}\times F_{2}=\left\{{(F}_{ij}^{1},\;F_{lm}^{2}\right)\left|1\leq i,l\leq K,\;1\leq j,m\leq N\right\}$ consists of pairs of feature vectors such that a pair belongs to match class (y = 1) if both fingerprints belong to the same subject and the class of the pair is non-match (y = 0) otherwise. The number of non-match pairs is much bigger than that of match pairs. The problem is to learn a metric $d:F_{1}\times F_{2}\to \;Y$, where Y={0,1}, y=1 means match class and y=0 means non-match class, such that

(1) $$\;S\left(d{(F}_{ij}^{1},\;F_{lm}^{2}\right))=\{\begin{array}{cc} \;1 & if\;d{(F}_{ij}^{1},\;F_{lm}^{2})\;<T\;thatis,\;F_{ij}^{1}\;and\;F_{lm}^{2}\;belong\;to\;the\;same\;finger\\ \;0 & otherwise, \end{array}$$

where *S(.)* is a threshold function, which takes distance $d{(F}_{ij}^{1},\;F_{lm}^{2})$ as input, and the threshold T∈(0,1) is a tradeoff between the true acceptance rate and false rejection rate. The metric function d can be modeled in different ways. Keeping in view the superior performance of deep learning in various computer vision problems [19,20], we model it using Siamese network with convolutional neural network as backbone model, and use adversarial learning for its training. The detail of this network is given in following sections.

### 2.2. Siamese Network for Matching

**Figure 2:**
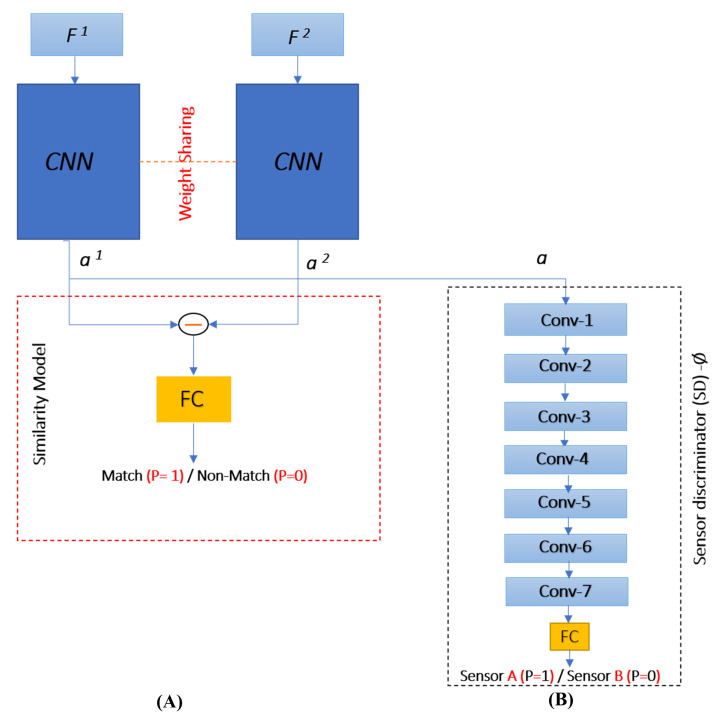
The design of the (**A**) Siamese network for matching (SiameseFinger) and (**B**) sensor discriminator (SD).

For simplicity, we denote the feature vectors of two fingerprints to be matched as $F^{1}$ and $F^{2}$. The matching of two fingerprints using their feature vectors $F^{1}$ and $F^{2}$ is modeled as a Siamese network, the high-level design of the network is shown in Figure 2. It consists of three main components: two CNN models, which have the same architecture, and one similarity module. The Siamese network plays the role of a generator in adversarial learning settings and another CNN model, shown in Figure 2, acts as a discriminator. The details of the backbone CNN model, the similarity module and adversarial learning are given in the following subsections.

#### 2.2.1. The Backbone CNN Model

**Figure 3:**
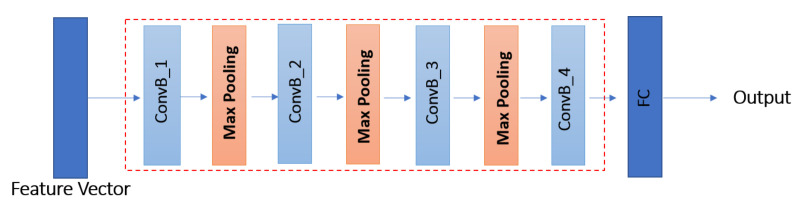
Linear convolutional neural network model used a backbone in Siamese network.

**Figure 4:**
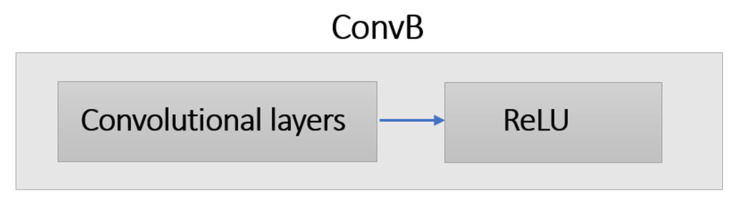
The detail of the layers in each convolutional block (ConvB).

Different architectures of the CNN model are possible based on two main design choices: Linear and DAG. In linear design, different layers; such as Convolutional layer (Conv), Rectified linear unit activation (ReLU), Fully connected layer (FC) and so forth; are stacked in such a way that the output of a lower level layer is passed as an input to the next higher level layer. AlexNet [19] and VGGNet [21] are two examples of CNN models based on linear design. Motivated by the simplicity and the success of linear models, we build a simple linear CNN model for Siamese network, in which we determined the architecture of the CNN model through experiments as shown in Figure 3. It consists of four Conv blocks, three pooling layers, and one fully connected (FC) layer. Pooling layers reduce the dimensionality of the input space and help to avoid overfitting by reducing the number of parameters. Each Conv block (ConvB) consists of two layers: Conv, and ReLU as shown in Figure 4.

The first Conv block ConvB_1 takes one dimensional (1D) feature vector $F^{i}\left(i=1,\;2\right)$ of size 4 × 648 as input and consists of 64 filters of size 1 × 10, stride 1, and no boundary padding. The second Conv block ConvB_2 contains 128 filters of size 1 × 7, and stride 1, and the third Conv block ConvB_3 contains 128 filters of size 1 × 4 and stride 1, and the fourth Conv block ConvB_4 contains 256 filters of size 1 × 5 and stride 1. For each Conv block, we employ ReLU activation because it results in fast convergence [19]. Each pooling layer applies max pooling operation with a window of size 1×2, and stride 2. The last layer is an FC layer which consists of 512 neurons. The output $a^{i}\left(i=1,\;2\right)$ of each CNN model in the Siamese network is passed to the similarity module as well as the sensor discriminator, which discriminates whether the fingerprint is captured with sensor A or sensor B.

Similarly, we determined the architecture of the sensor discriminator based on our experiments. It is modeled with a CNN model, which consists of seven Convolutional layers without using ReLU layers and max-pooling layers. The first Conv layer Conv_1 consists of 64 filters, and each of the other Conv_2, Conv_3, and Conv_4 consists of 128 filters, Conv_5 and Conv_6 consist of 256 filters, the last Conv_7 consists of 512 filters and all these Conv layers consist of filters of size 1 × 1 and stride 1. The last layer is an FC layer which consists of one neuron.

#### 2.2.2. Similarity Module

The first block in the similarity module is difference layer that takes two features maps $F^{1}$ and $F^{2}$ as inputs, please see Figure 2A. It computes the channel-wise difference between the feature maps $F^{1}$ and $F^{2}$. In this way, it determines whether the corresponding features in each fingerprint are similar or not. The output of the difference layer is passed to an FC layer, which learns the discriminative features and performs the classification to yield the prediction of whether the two fingerprints are matching or non-matching. If the pair matches, it belongs to the same class (*p* = 1), otherwise it belongs to non-match class (*p* = 0).

#### 2.2.3. Loss Functions for Training the Network

To ensure the robust matching results, the network is trained so that the learned features have high inter-class variation and low intra-class discrimination. For this purpose, we employ the commonly used loss function: binary-class cross-entropy (BCE) loss. Without loss of generality, we assume that $B\subseteq F_{1}\times F_{2}$ is a mini-batch of size *b* such that $B=\{P_{r}|P_{r}=\left(F_{i_{r}j_{r}}^{1},\;F_{l_{r}m_{r}}^{2}\right),1\leq r\leq b\}$, and $Y_{B}=\left\{y_{r}|y_{r}=1\;or\;0,\;1\leq r\leq b\right\}$. is the corresponding set of labels so that $y_{r}=1$ for match pair $P_{r}$ and vice versa. Let $d\left(P_{r}\right)=d\left(F_{i_{r}j_{r}}^{1},\;F_{l_{r}m_{r}}^{2}\right)=p_{r},\left(0\leq p_{r}\leq 1\right)$ be the prediction of the rth pair $P_{r}$. The BCE loss is defined as follows:

(2) $$L_{bce}\left(\theta \right)=-\frac{1}{b}\sum_{r=1}^{b}y_{r}logp_{r}+{(1-y}_{r})log{(1-p}_{r}),$$

where θ represents the parameters of the Siamese network. For a matching pair (or non-match pair), if it is wrongly predicted as non-match pair (or match pair), it adds big value to the loss and causes the parameters to be updated through backpropagation so that the loss is reduced that is, each type of pair is correctly classified. In this way, this loss function forces the network to learn the features which have high inter-class variation and low intra-class discrimination so that each type of pair is correctly classified.

### 2.3. Adversarial Learning and Sensor Discriminator

To train the network, we use adversarial learning, which performs learning using a mini-max game between two players, the generator and the discriminator; the discriminator acts as adversary. The proposed Siamese network is treated as a generator and for discriminator, a network is introduced. The role of the discriminator is to discriminate whether the fingerprint is captured with sensor A or sensor B, we call it sensor discriminator (SD), and denote it with ϕ. The design of the network in adversarial learning setting is shown in Figure 2B, and the detail of its architecture is given in Section 2.2. The SD is a CNN model that takes as input the activation *a* (corresponding to sensor A or sensor B) from the Siamese network and predicts its label as sensor A (*l* = 1) or B (*l* = 0) that is, it is a binary classifier that maps *a* to the probability $p(\phi \left(a\right)=p)$, where *p* is the probability that *a* is from sensor A or sensor B; if p≈1, then the fingerprint is from sensor A, otherwise it is from sensor B (p≈0). The SD tries to minimize the adversarial loss defined as follows:

(3) $$L_{sd}\left(\theta_{sd}\right)=-\frac{1}{b}\sum [l\;log\;p+\left(1-l\right)\log\left(1-p\right),]$$

which is the loss of the SD predictions corresponding to all fingerings in the mini-batch B. The training of the Siamese network is carried out by jointly minimizing the losses $L_{bce}\left(\theta \right)$ and $L_{sd}\left(\theta_{sd}\right)$ as a mini-max game [22] with the following processes:

(4) $$\hat{\theta }=\min\;\theta \left(L_{bce}\left(\theta \right)-L_{sd}\left({\hat{\theta }}_{sd}\right)\right)$$

(5) $${\hat{\theta }}_{sd}=\max\;\theta_{sd}\left(L_{bce}\left(\theta \right)-L_{sd},\left(\theta_{sd}\right)\right)$$

that is, the Siamese network learns its parameter θ by minimizing its loss and increasing the loss of SD concurrently and vice versa. In other words, the Siamese network updates its parameters to generate the features, which can correctly classify the input pair as match or non-match, but SD cannot discriminate whether the fingerprint is from sensor A or B based on these features. On the other hand, the SD updates its parameters so that it can correctly identify the sensor type. This mini-max game between the Siamese network and the SD helps the Siamese network to learn the discriminative features with high inter-class discrimination and low intra-class variation for the match and non-match classification of fingerprint pairs. After computing the losses, the gradient is updated by minimizing the loss of the generator and maximize the loss of the discriminator, concurrently and vice versa. This minimax game is performed using a stochastic gradient descent optimization algorithm. To update discriminator parameters and maximize the discriminator loss we use Gradient Reversal Layer (GRL) [23], which reverses the gradient direction by multiplying it by a negative scaler during the backward pass.

## 3. Evaluation Protocol

First, we give a detail of the databases, which were used to evaluate the effectiveness of the proposed system. Then, the evaluation procedure and the detail about the training of the Siamese network are presented.

### 3.1. Description of Datasets

**Figure 5:**
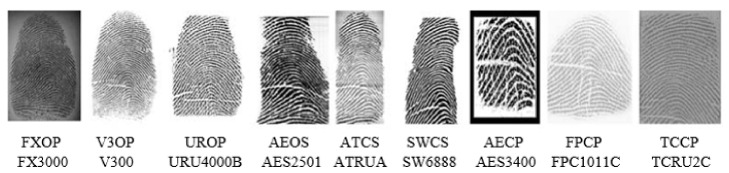
Example Fingerprints of the same finger from FingerPass Database. The first row indicates the name of the dataset name and the second-row the sensor name.

**Figure 6:**
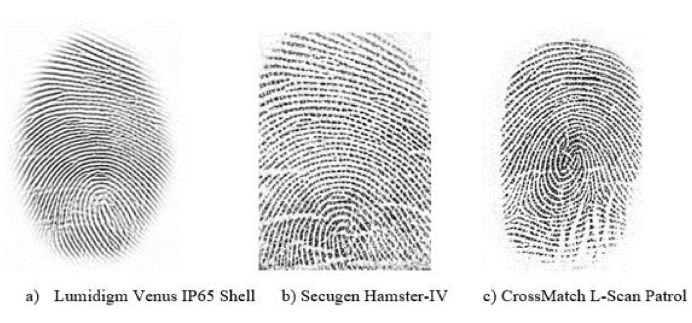
Example fingerprints of the same finger captured by 3 optical sensors from MOLF database.

Databases are very essential for evaluating and analyzing the performance of the proposed system. However, only a few public benchmark databases exist for the fingerprint sensor interoperability problem such as FingerPass [12] and MOLF [13], which consists of fingerprints that were captured with different sensors having different technology and interaction types. The FingerPass was collected from 9 different sensors and it contains 9 sub-databases. It contains 720 individual fingerprints with 12 impressions each. So, the total number of fingerprints is 720 × 12 × 9 = 77,760. Table 1 summarizes the detail of FingerPass datasets. The FingerPass datasets are divided into nine groups, as shown in Table 1, according to types of sensors (optical and capacitive) and interaction types (press and sweep), some fingerprints from these datasets are shown in Figure 5. MOLF [13] database is an other benchmark cross-device database that includes three datasets captured using three optical sensors. It contains 12,000 fingerprints captured from 10 fingers of 100 subjects; four impressions were obtained from each finger with three sensors each, so the total number of fingerprints is 10 × 100 × 4 × 3 = 12,000. The number of fingerprints from each sensor is 4000. The resolutions of the fingerprints acquired from the three sensors are 352 × 544, 258 × 336, and 1600 × 1500 pixels; These datasets are referred to as DB1, DB2, and DB3. Figure 6 shows some fingerprints from MOLF database.

### 3.2. Evaluation Procedure

The proposed method is evaluated using the well-known evaluation metric—equal error rate (EER) [24]. The EER is the operating point at which the false non-match rate (FNMR) and false match rate (FMR) are equal. FMR is the rate at which the matching algorithm accepts the fingerprints from different subjects as the fingerprints of the query subject while FNMR is the rate at which the matching algorithm rejects to take the fingerprints from the query subjects to be his/her fingerprints. We employed two matching scenarios to evaluate the proposed method: (1) Regular matching, two fingerprints captured by the same sensor are compared for verification and the performance metric is known as a native-EER); and (2) Cross-matching, in this case, two fingerprints captured using different sensors are compared and the performance metric is referred to as cross-EER or interoperable-EER. For evaluating the matching, we adopt the same protocol as was used in [12], in which the data is divided into gallery and probe datasets, and impostor match scores and genuine match scores are computed. The proposed model is trained using the FingerPass and MOLF datasets, independently. For cross-sensor matching on FingerPass, the datasets are arranged into pairs; one dataset is taken as galley set and the other dataset is treated as probe set. Each dataset of FingerPass contains 12 impressions of each of 720 fingers (total 8640 fingerprints). We split the probe set into training, validation and testing sets in such a way that 8 impressions from each finger are used for training the model (5760 fingerprints), 1 for validation (720 fingerprints), and 3 for testing (2160 fingerprints). Using the training fingerprints from probe set and the gallery set, we prepare match and non-match pairs of fingerprints for training. Similarly, we prepare match and non-match pairs for validation and testing. In MOLF dataset, each finger has 4 impressions, so, we took 2 impressions for training the model (2000 images), 1 for validation (1000 images), and 1 for testing (1000 images).

### 3.3. Training Model

Before passing the features to the Siamese network, they are preprocessed using min-max normalization [25], to speed up the training process. We employed ADAM for training the network. In our experiments, we set the learning rate to 6 × 10${}^{-5}$, the total number of epochs is five, and each epoch takes 1000 iterations with a minimum batch of 300 match and non-match pairs. As the number of non-match pairs is larger than that of match pairs, so to overcome the problem of imbalance data, we selected randomly the equal number of match and non-match pairs for each batch.

## 4. Experimental Results and Discussion

In this section, first we present the results to assess the performance of the proposed method on two databases. Then, we compare its performance with that of the state-of-the-art methods.

### 4.1. Experimental Results on the FingerPass Database

**Figure 7:**
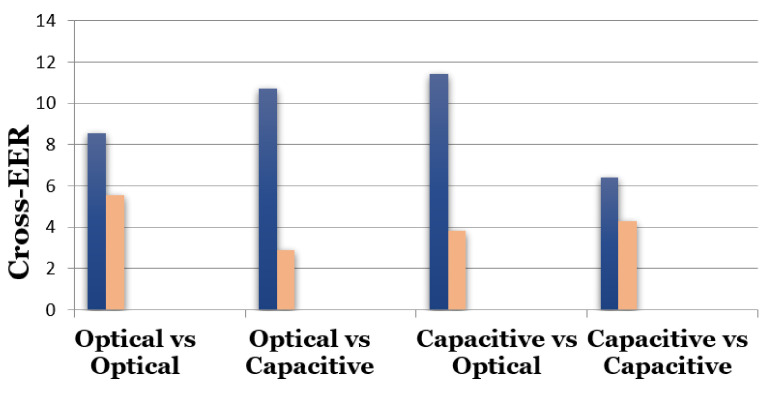
Maximum (blue) and minimum (pink) cross-EER on nine datasets of the FingerPass database.

Table 2 presents the verification results of the proposed method in terms of EER as well as the accuracy on AEOS, AECP, FPCP, SWCS, TCCP, V3OP, ATCS, FXOP and UROP datasets from the FingerPass database. Accuracy is computed as the percent of test match and non-match pairs, which were correctly classified. The native-EER (when the same sensor is used for probe and gallery) is comparatively small and less than 3 for all datasets and the accuracy is high except UROP, V3OP and AECP, where EER is slightly higher than 3 and accuracies are higher than 96%. Furthermore, it can be observed that the native-EER is much smaller when AEOS, SWCS, ATCS and FPCP datasets are used. The best performance is obtained (ATCS with native-EER of 0.998) when the technology type is capacitive and interaction type is sweep and the image resolution is 500 dpi. However, when the interaction type is press, the native-EER is usually higher. The results in Table 2 indicate that overall cross-EER is higher than native-EER. The EER is the highest when UROP is gallery set and TCCP or AECP is probe set (optical vs capacitive), with different image resolutions of 700 dpi (UROP) and 500 dpi (TCCP); in both cases the interaction type is press. The cross-EER values vary from 11.433 to 2.869 when different gallery and probe sets are used, depending on the technology type, interaction type and the image resolutions. The minimum cross-EER is 2.869 when AEOS is used as gallery set and SWCS is used as prob set (optical vs capacitive), in this case, both sets have the same image resolution 500 dpi and the same interaction type that is, sweep. The cross-EER is maximum that is, 11.433 (10.700) when AECP (V3OP) is used as gallery set and V3OP (AECP) is used as prob set. The reason for this high cross-EER is the big difference in the sizes of fingerprints; in case of AECP the image size is 144×144, whereas it is 640×480 in case of V3OP. Figure 7 shows the maximum (blue) and minimum (pink) cross-EER when different sets of the FingerPass database are used as gallery and prob sets. It can be observed that if the two datasets (optical vs. capacitive) are acquired with sensor of press interaction type with a resolution higher than 500 dpi, the cross-EER becomes slightly high and if the same two datasets are from sweep sensor with a resolution of 500 dpi, the cross-EER becomes slightly less.

### 4.2. Experimental Results on The MOLF Database

The results on the three datasets (DB1, DB2, and DB3) from the MOLF database are presented in Table 3. Overall the native-EER is small and it is minimum for the DB1 dataset and maximum for the DB2 dataset. In cross-matching cases, the cross-EER is significantly higher than native-EER. One possible reason for higher EER is that the number of fingerprints is small in this case that is, only two fingerprints per finger are available for training the Siamese network.

### 4.3. Comparisons with the State-of-the-Art Methods

To evaluate the performance of the proposed method, the results are compared with the state-of-the art cross-sensor matching methods: MCC [26], VeriFinger [27], CrossVFinger [4] employing Gabor-HoG only.

#### 4.3.1. Results on the MOLF Database

The SiameseFinger achieves good performance in case of regular matching compared to CrossVFinger with Gabor-HoG descriptor. For cross-matching, the performance of SiameseFinger is comparable with that of CrossVFinger. The SiameseFinger achieves low cross-EER for the cases DB2 vs DB1 and DB1 vs DB2.

**Figure 8:**
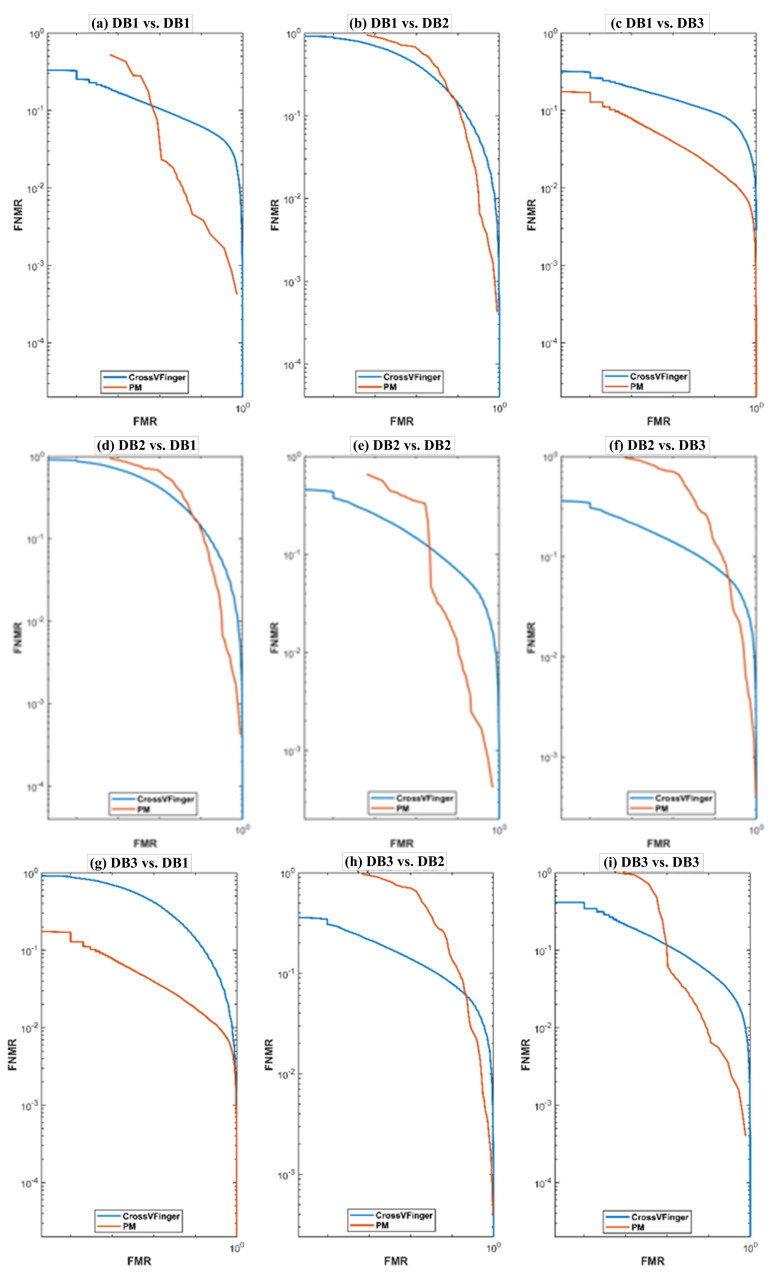
DET curves corresponding to SiameseFinger (PM) and CrossVFinger on the MOLF database: (**a**) DB1 vs. DB1, (**b**) DB1 vs. DB2, and (**c**) DB1 vs. DB3, (**d**) DB2 vs. DB1, (**e**) DB2 vs. DB2, and (**f**) DB2 vs. DB3, (**g**) DB3 vs. DB1, (**h**) DB3 vs. DB2, and (**i**) DB3 vs. DB3.

Figure 8 shows the DET curves for CrossVFinger and the SiameseFinger. The DET curves are almost in agreement with the results in Table 4. The SiameseFinger outperforms CrossVFinger. In almost all cases, when FMR of SiameseFinger (PM) increases, there is a faster decrease in its FNMR as compared to that of CrossVFinger.

#### 4.3.2. Results on The FingerPass Database

We compared the performance of SiameseFinger on six datasets of the FingerPass database: AECP, AEOS, ATCS, SWCS, FPCP, and UROP that is, two optical and four capacitive sensors with three state-of-the-art methods: MCC [26], Verifinger [27] and CrossFinger [4]. The results of the comparison are shown in Table 5.

Overall, SiameseFinger outperforms MCC, VeriFinger and CrossVFinger in all cases except in the native matching and one cross-matching case (UROP vs. AECP). In the case of native matching, PM gives a better performance than MCC and verifinger on FPCP and AECP. MCC is based on a fixed-length and discriminative type of 3D data structures, called cylinders, constructed from minutia angles and distances; as the minutia angles and distances are dependent on the distortion of fingerprints, so the descriptors of the fingerprints of the same finger captured with different sensors are different because different sensors introduce different distortions; because of this, the performance of MCC is very poor. VeriFinger uses minutia points as well as extra information such as ridge count. The experimental results described in Table 5 show that these methods are not robust for cross-matching problem. On the other hand, CrossVFinger designed by taking into consideration the structural patterns that are not affected by sensor type but still not very effective for the interoperability problem. The SiameseFinger yields better cross-matching performance than MCC, VeriFinger, and CrossVFinger. Overall, the best matching performance was achieved in optical vs. capacitive scenarios. The SiameseFinger performs better than other methods due to two reasons. First, it employs a convolutional neural network as a backbone model, which learns the discriminative information by analyzing hierarchically the information from both fingerprints. Second, it learns using adversarial learning, which guides the Siamese network to learn the features which have low intra-class variations and high inter-class scatter. Siamese network for matching is better than simple deep neural networks because it learns the discriminative features by comparing the probe and gallery fingerprints.

### 4.4. Model Complexity

The SiameseFinger was implemented in the MATLAB (R2020b) environment and for the experiments, we use a PC (Intel Core i9-7900X CPU @ 3.30 GHz–3.31 GHz, 64.0 GB RAM) and Microsoft Windows 10 in the 64-bit operating system, x64-based processor. Table 6 shows a comparison of SiameseFinger, CrossVFinger [4] and Verifinger [27]. It can be noted that the matching time of SiameseFinger is longer than the CrossVFinger [4] and less than Verifinger [27].

## 5. Conclusions

In this paper, we introduced a cross-matching system SiameseFinger to minimize the effect of the cross-sensor fingerprint matching. The SiameseFinger is based on deep learning as a new method to reduce the effect of the interoperability problem. The method uses the Siamese network to learn the hierarchy of features so that it can correctly classify the input pair as matching or non-matching. This Siamese network uses the Gabor-HoG descriptor features as an input and we adapt a convolutional neural network as a sensor discriminator for adversarial learning. Several experiments have been carried out on two benchmark databases: FingerPass and MOLF, which focus on the fingerprint sensor interoperability problem, and we used the well-known metric (EER) to evaluate SiameseFinger in two matching scenarios: regular matching (native-EER), and cross-matching (cross-EER). Intensive experiments were performed to evaluate the performance of SiameseFinger and it was compared with the state-of-the-art methods. The results show that SiameseFinger significantly outperformed these methods. One limitation of SiameseFinger is that its performance drops when sizes of gallery and probe fingerprints differ significantly. In future work, we aim to address this problem and introduce a new method for feature fusion for cross-sensor matching based on adversarial learning to further improve the cross-sensor performance.

## Figures and Tables

**Table 1:** Detail of FingerPass datasets [12]. The first two characters in the name of each dataset indicate the sensor type, whereas the last two characters represent the technology type (O-optical, C-capacitive) and the interaction type (P-press, S-sweep), respectively.

Dataset	Sensor	Technology Type	Interaction Type	Image Size (Pixels)	Image Resolution
FXOP	FX3000	Optical	Press	400 × 560	569 dpi
V3OP	V300	Optical	Press	640 × 480	500 dpi
UROP	URU4000B	Optical	Press	500 × 550	700 dpi
AEOS	AES2501	Optical	Sweep	Variable	500 dpi
ATCS	ATRUA	Capacitive	Sweep	124 × 400	250 dpi
SWCS	SW6888	Capacitive	Sweep	288 × 384	500 dpi
AECP	AES3400	Capacitive	Press	144 × 144	500 dpi
FPCP	FPC1011C	Capacitive	Press	152 × 200	363 dpi
TCCP	TCRU2C	Capacitive	Press	208 × 288	500 dpi

**Table 2:** The results of SiameseFinger on nine datasets from the FingerPass database.

Gallery Dataset	Probe Dataset	EER	Accuracy
**Native-Matching**
UROP	UROP	3.509	96.63%
TCCP	TCCP	2.646	97.54%
AEOS	AEOS	1.489	98.53%
SWCS	SWCS	2.242	97.89%
FPCP	FPCP	1.995	98.12%
AECP	AECP	3.738	96.33%
ATCS	ATCS	0.998	98.97%
V3OP	V3OP	5.167	94.77%
FXOP	FXOP	2.638	97.43%
**Cross-Matching**
UROP	TCCP	7.175	92.61%
UROP	AECP	7.177	92.56%
UROP	FPCP	5.923	94.03%
UROP	AEOS	5.555	94.35%
UROP	SWCS	5.694	94.43%
FPCP	UROP	6.801	93.23%
FPCP	AEOS	3.843	96.14%
TCCP	AEOS	4.259	95.19%
AECP	AEOS	5.752	94.19%
TCCP	SWCS	4.305	95.74%
AECP	SWCS	6.426	93.86%
AECP	ATCS	4.694	95.52%
FPCP	TCCP	5.740	94.67%
AEOS	SWCS	2.869	96.99%
AEOS	FPCP	4.583	95.52%
ATCS	AECP	6.111	93.95%
AECP	FXOP	8.840	91.66%
FXOP	AECP	8.853	91.35%
V3OP	AECP	10.700	90.12%
AECP	V3OP	11.433	90.71%
V3OP	FPCP	7.591	92.23%
FPCP	V3OP	8.240	91.89%
V3OP	FXOP	7.601	92.50%
FXOP	V3OP	8.555	90.75%

**Table 3:** The results of SiameseFinger on MOLF database in terms of EER.

Gallery/Probe	DB1	DB2	DB3
DB1	1.83 (98.16%)	10.47 (82.16%)	10.48 (81.56%)
DB2	10.23 (83.90%)	3.52 (96.02%)	10.29 (82.02%)
DB3	10.73 (80.18%)	10.3 (82.82%)	2.69 (96.78%)

**Table 4:** Comparsion of the SiameseFinger with CrossVFinger on the MOLF database in terms of EER.

Gallery/Probe	SiameseFinger	CrossVFinger (Gabor-HoG)
	DB1, DB2, DB3	DB1, DB2, DB3
DB1	1.83, 10.47, 10.48	6.80, 11.81, 9.93
DB2	10.23, 3.52, 10.29	11.81, 7.48, 8.79
DB3	0.73, 10.3, 2.69	9.93, 8.79, 6.59

**Table 5:** Comparison of the performance of SiameseFinger with the state-of-art methods on FingerPass database in terms of EER.

Gallary	FPCP	AECP	SWCS	AECP	UROP	FPCP	AECP	UROP	AEOS	ATCS	AECP	FPCP
**Probe**	**FPCP**	**AECP**	**SWCS**	**AEOS**	**FPCP**	**AEOS**	**SWCS**	**AECP**	**FPCP**	**AECP**	**ATCS**	**UROP**
MCC	25.37	43.18	3.07	34.71	46.44	41.25	36.88	43.98	41.25	47.69	47.7	46.44
Verifinger	5.2	12.87	0.45	10.62	43.3	28.99	12.81	27.81	28.98	30.32	30.33	43.35
CrossVFinger	0.754	0.578	0.002	6.543	6.829	6.872	6.427	5.565	6.872	6.717	6.717	6.82
SiameseFinger	1.955	3.738	1.92	5.752	5.923	3.843	6.426	7.177	4.583	6.111	4.694	6.8

**Table 6:** Comparison of SiameseFinger, CrossVFinger [4] and Verifinger [27].

Model	Matching Time
CrossVFinger [4]	0.000143 s
Verifinger [27]	0.794 s
SiameseFinger	0.01881 s

## Data Availability

The MOLF dataset is available at: http://research.iiitd.edu.in/groups/iab/molf.html and the FingerPass (accessed on 22 May 2021) dataset is available at: http://www.fingerpass.csdb.cn/ (accessed on 22 May 2021).
